# The burden of headache following aneurysmal subarachnoid hemorrhage: a prospective single-center cross-sectional analysis

**DOI:** 10.1007/s00701-020-04235-7

**Published:** 2020-02-04

**Authors:** Torge Huckhagel, Regine Klinger, Nils Ole Schmidt, Jan Regelsberger, Manfred Westphal, Patrick Czorlich

**Affiliations:** 1 Department of Neurosurgery, University Medical Center Hamburg-Eppendorf, Martinistraße 52, 20246 Hamburg, Germany; 2 Department of Anesthesiology, University Medical Center Hamburg-Eppendorf, Martinistraße 52, 20246 Hamburg, Germany

**Keywords:** Headache, Intracranial aneurysm, Quality of life, *Subarachnoid hemorrhage*

## Abstract

**Background:**

Aneurysmal subarachnoid hemorrhage (SAH) as a serious type of stroke is frequently accompanied by a so-called initial thunderclap headache. However, the occurrence of burdensome long-term headache following SAH has never been studied in detail so far. The aim of this study was to determine the prevalence and characteristics of long-term burdensome headache in good-grade SAH patients as well as its relation to health-related quality of life (HR-QOL).

**Methods:**

All SAH cases treated between January 2014 and December 2016 with preserved consciousness at hospital discharge were prospectively interviewed regarding burdensome headache in 2018. Study participants were subsequently scrutinized by means of a standardized postal survey comprising validated pain and HR-QOL questionnaires. A retrospective chart review provided data on the initial treatment.

**Results:**

A total of 93 out of 145 eligible SAH patients participated in the study (62 females). A total of 41% (38/93) of subjects indicated burdensome headache at follow-up (mean 32.6 ± 9.3 months). Comparison between patients with (HA+) and without long-term headache (HA-) revealed significantly younger mean age (47.9 ± 11.8 vs. 55.6 ± 10.3 years; *p* < .01) as well as more favorable neurological conditions (WFNS I/II: 95% vs. 75%; *p* = .03) in HA+ cases. The mean average headache of the HA+ group was 3.7 ± 2.3 (10-point numeric rating scale), and the mean maximum headache intensity was 5.7 ± 2.9. Pain and HR-QOL scores demonstrated profound alterations in HA+ compared to HA- patients.

**Conclusions:**

Our results suggest that a considerable proportion of SAH patients suffers from burdensome headache even years after the hemorrhage. Moreover, long-term headache is associated with reduced HR-QOL in these cases.

**Electronic supplementary material:**

The online version of this article (10.1007/s00701-020-04235-7) contains supplementary material, which is available to authorized users.

## Introduction

Aneurysmal subarachnoid hemorrhage **(SAH)** is a frequently devastating condition which constitutes about 5% of all strokes with a variable annual incidence between 0.7 and 23.9 cases per 100,000 inhabitants among different regions of the world with slight decline over the past decades [16, 29, 56, 57]. Despite of its relatively rare occurrence, SAH contributes significantly to stroke-associated reduction of health-related quality of life, morbidity, and mortality mainly due to the severity of the condition and the young average age of the affected patients [4, 9, 43, 66]. A decrease in case fatality rates could be determined over the past decades due to improved therapeutic management, but the long-term health-related quality of life **(HR-QOL)** of these patients resulting from physical, cognitive, and psychological impairment as well as associated medical conditions like chronic headache has not been sufficiently elucidated so far [2, 31, 37, 41, 56]. This study aims to characterize the long-term burden of headache and its influence on HR-QOL after aneurysmal subarachnoid hemorrhage.

## Methods and materials

Protocol and patient consent forms of this prospective single-center cross-sectional investigation were approved by the Ethics Committee of the Hamburg Medical Council (reference number PV5584). The study was conducted in accordance with the Declaration of Helsinki adopted by the World Medical Association General Assembly in 1964 and its later amendments. Study conception, data collection, and presentation strictly adhere to the principles of good practice in the conduct and reporting of survey research proposed by Kelley et al. in 2003 [35].

A total of 145 patients treated for newly diagnosed SAH at our tertiary referral center between January 2014 and December 2016 fulfilled the beforehand specified eligibility criteria of an age of 18 years or older and favorable neurological status defined as Glasgow Outcome Scale Score (**GOS)** ≥ 3 at hospital discharge [33]. All cases were either direct admissions or early transfers from surrounding clinics within the catchment area of our institution. The diagnosis of SAH was based on cranial computed tomography and/or magnetic resonance imaging and/or lumbar puncture. A four-vessel digital subtraction angiography was utilized to determine the location and morphological aspects of the underlying aneurysm in all cases. Patients suffering from hemorrhages due to non-aneurysmal pathologies were excluded. To improve the overall participation rate and reduce the bias effects of non-response, all patients or their legal guardians were contacted by phone in advance and informed briefly about the scope and expected time requirement in case of voluntary study participation. All telephone interviews were performed personally by the principal investigator (TH). After exclusion of 52 cases due to various reasons set out in Flowchart 1**,** questionnaires including detailed study descriptions and informed consent forms were sent by mail to all 93 patients (31 males, 62 females) willing to take part in the survey in 2018 (mean follow-up after SAH 32.6 months ±9.3 SD).

**Flowchart 1:**
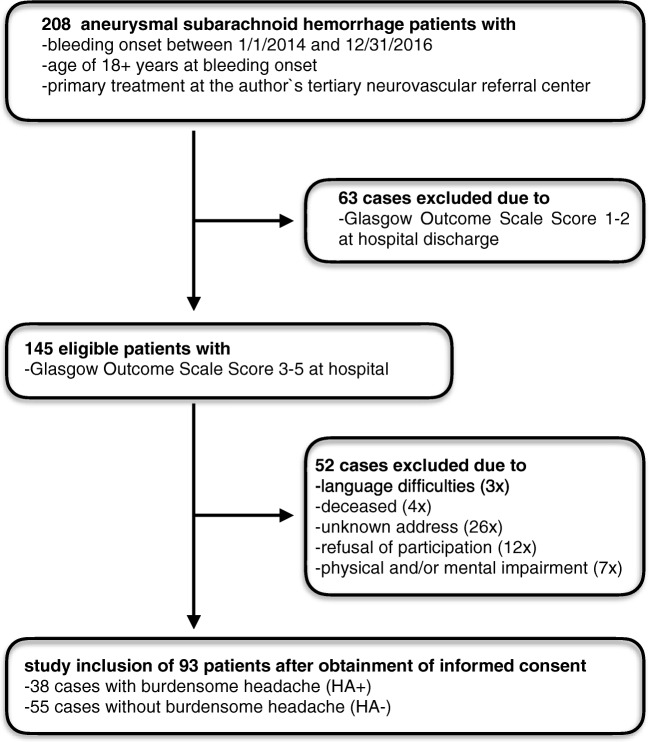
Study inclusion criteria and reasons for exclusion of potentially eligible cases

We used the German version of the 12-Item Short-Form Health Survey **(SF-12)** as a well-established generic measure of physical and mental health [67]. Multidimensional headache assessment was achieved by the combined implementation of the German pain questionnaire **(DSF)** of the German Society for the Study of Pain containing among other tools a 10-point numeric pain rating scale **(NRS)** ranging from 0 (=no pain) to 10 (maximum pain) and the depression, anxiety, and stress scale **(DASS)** [21, 47, 49], the short-form McGill Pain Questionnaire **(SF-MPQ),** and the German version of the Henry Ford Hospital Headache Disability Inventory **(HDI-G)** [6, 32, 44].

All patients were initially asked whether they suffered from continuous or recurrent burdensome headache for 6 months or longer at the time of the interview. This kind of headache differs from the so-called headache attributed to SAH (International Classification of Headache Disorders of the International Headache Society 2nd Edition Code 6.2.2) which develops, by definition, simultaneously with the bleeding and resolves within 1 month thereafter [26]. According to their reply to the previous question, all cases were separated into two groups: SAH with **(HA+;** n **= 38)** or without **(HA-;** n **= 55)** burdensome headache.

Additionally, clinical and radiographic data concerning the initial inpatient course was obtained from the electronic medical records of our institution. Clinical information comprises the epidemiological data, aneurysm features including size and location (by definition, anterior circulation consists of the following four vessels: internal carotid artery, middle cerebral artery, anterior cerebral, and anterior communicating artery), SAH severity was defined using the World Federation of Neurological Surgeons Grading **(WFNS)** [50], the latter of whom containing the Glasgow Coma Scale as main component [63], treatment mode (endovascular vs. microsurgical clipping), hospital and intensive care unit **(ICU)** length of stay as well as functional outcome **(GOS).** Fisher, Graeb, and Hijdra Scores were utilized to quantify the amount of subarachnoid and intraventricular blood [18, 23, 27].

All clinical and radiographic parameters as well as questionnaire-derived scores were calculated separately for both collectives and group comparisons were performed. Variables are presented utilizing descriptive statistics, inter alia, fractions, central tendency (mean/median), and appropriate dispersion measures (e.g., standard deviation (SD)). Fisher’s exact test was applied with regard to categorical variables, and Mann-Whitney test as well as unpaired t-test was utilized depending on whether non-parametric or parametric data was available for comparison. Multivariate logistic regression was performed to determine the predictive value of epidemiological and clinical variables showing a *p* value < 1 in the univariate analysis (patient age at bleeding onset, initial WFNS grade, and GOS score at discharge) with regard to headache occurrence at follow-up. Results are displayed in the form of odds ratios **(OR)** with 95% confidence intervals **(CI).** All statistical procedures were carried out with GraphPad Prism (version 7.0, GraphPad Software, La Jolla California, USA) and SPSS (version 22.0, International Business Machines Corporation (IBM), Armonk, USA). A *p* value < 05 was considered to indicate statistical significance. A biostatistician was involved in the planning and realization of all statistical procedures.

## Results

### Epidemiology, clinical, and radiographic SAH characteristics

Mean age, sex, and clinical WFNS scores of SAH patients did not differ significantly between the study participants (*n* = 93), and non-responders (*n* = 52). HA+ (*n* = 38) and HA- (*n* = 55) patients presented without significant gender disparities, whereas the headache group was of markedly younger average age in comparison to their counterparts (mean ± SD (years): 47.9 ± 11.8 versus 55.6 ± 10.3; *p* < .01). WFNS grade distribution was notably divergent in both collectives with HA+ showing better WFNS scores (WFNS I/II in 95% (HA+) and 75% (HA-); *p* = .03). No significant differences between both cohorts could be determined regarding body mass index, initial transient loss of consciousness, maximum intracranial pressure (<24 h post bleeding) a hyperactive delirium during ICU treatment period, as well as GOS at discharge (GOS IV/V in 100% (HA+) versus 76% (HA-); *p* = .08). Considering Fisher, Graeb, and Hijdra scores as well recognized SAH radiographic features, none of them revealed significant differences between both categories. Comprehensive information on all epidemiological, clinical, and radiographic data discussed here is provided by Table 1.

**Table 1:** Epidemiological, clinical, and radiographic characteristics of SAH patients with and without burdensome headache

	SAH with headache	SAH without headache	SAH total	Significance
Mean age/SD (years)	47.9/11.8	55.6/10.3	52.5/11.5	p < .01*
Sex ratio (male/female)	10/28 (26.3%/73.7%)	21/34 (38.2%/61.8%)	31/62 (33.3%/66.7%)	p = .27**
Mean body mass index/SD	24.9/4.5	25.6/5.0	25.3/4.8	p = .52*
Initial unconsciousness	8/38 (21.1%)	18/55 (32.7%)	26/93 (28.0%)	p = .25**
Hyperactive delirium	19/35 (54.3%)	31/52 (59.6%)	50/87 (57.5%)	p = .66**
Mean highest ICP (< 24 h)/SD (mmHg)	12.4/4.6	11.0/3.6	11.5/4.0	p = .24*
Median WFNS grade/25%–75% interval	1/1–1	1/1–3	1/1–1	p = .03***
Median Fisher grade/25%–75% interval	4/3–4	4/3–4	4/3–4	p > .99***
Median Graeb score/25%–75% interval	2/0–3	1/0–4	1/0–4	p = .98***
Median Hijdra score/25%–75% interval	13/9–18	15/7–21	13/8–20	p = .51***
Intraparenchymal hemorrhage	7/38 (18.4%)	17/55 (30.9%)	24/93 (25.8%)	p = .23**
Median GOS/25%–75% interval	5/4–5	5/4–5	5/4–5	p = .08***

* Unpaired t-test. ** Fisher’s exact test. *** Mann-Whitney test. GOS = Glasgow Outcome Scale. ICP = intracranial pressure. SD = standard deviation. WFNS=World Federation of Neurological Surgeons Subarachnoid Hemorrhage Grading

### Aneurysm features and specification of SAH treatment

Both groups demonstrated no significant differences concerning side, location, and mean size of aneurysm as well as fraction of patients undergoing microsurgical clipping, temporary or permanent cerebrospinal fluid diversion via external ventricular drain, or subsequent ventriculoperitoneal shunt implantation. A total of 78.5% of bleedings resulted from an aneurysm originating from the anterior cerebral circulation. In total, 28.0% of all SAH cases were treated surgically by means of aneurysm clipping, and permanent cerebrospinal fluid drainage was necessary in 6.5% of all patients at discharge. Additionally, mean ventilation time, length of ICU, and total hospital stay exposed no significant distinctions between both collectives. Particulars regarding aneurysm and treatment details are presented extensively in Table 2.

**Table 2:** Aneurysm and treatment characteristics of SAH patients with and without burdensome headache

	SAH with headache	SAH without headache	SAH total	Significance
Anterior circulation aneurysm	29/38 (76.3%)	44/55 (80.0%)	73/93 (78.5%)	p = .80*
Mean aneurysm size/SD (mm)	5.5/2.6	6.2/3.5	5.9/3.2	p = .46**
Microsurgical clipping	9/38 (23.7%)	17/55 (30.9%)	26/93 (28.0%)	p = .49*
Temporary csf drain (EVD/LD)	20/38 (52.6%)	33/55 (60.0%)	53/93 (57.0%)	p = .53*
Ventriculoperitoneal shunt	1/38 (2.6%)	5/55 (9.1%)	6/93 (6.5%)	p = .40*
Mean ventilation time/SD (hours)	40.6/106.8	86.6/165.8	67.8/145.7	p = .29**
Mean total dexamethasone on ICU/SD (mg)	50.9/75.0	51.5/86.9	51.2/81.8	p = .60**
Mean length of ICU stay/SD (days)	17.6/10.7	18.1/10.3	17.9/10.4	p = .62**
Mean length of hospital stay/SD (days)	30.0/13.9	26.4/9.1	27.9/11.4	p = .35**

* Fisher’s exact test. ** Mann-Whitney test. EVD = external ventricular drainage. ICU = intensive care unit. LD = lumbar drainage. SD = standard deviation

### Comorbidities and early complications

At bleeding onset there were similar prevalences of neurological, psychiatric, cardiovascular, pulmonary, and other comorbidities as well as health-related lifestyle factors such as tobacco or alcohol consumption in both patient groups. Moreover, the rate of different SAH-related in-hospital complications, which are described together with the preexisting disorders in Table 3, revealed no significant differences between HA+ and HA-.

**Table 3:** Preexisting disorders and in-hospital complications of SAH patients with and without burdensome headache. Information on preexisting conditions were obtained from the SAH patient, next of kin or family physician

Disorder/complication	SAH with headache	SAH without headache	SAH total	Significance
Chronic headache condition	4/38 (10.5%)	7/55 (12.7%)	11/93 (11.8%)	p > .99*
Arterial hypertension	15/38 (39.5%)	19/55 (34.5%)	34/93 (36.6%)	p = .67*
Hypothyroidism	3/38 (7.9%)	7/55 (12.7%)	10/93 (10.8%)	p = .52*
Hyperlipidemia	4/38 (10.5%)	3/55 (5.5%)	7/93 (7.5%)	p = .44*
Diabetes mellitus	0/38 (0.0%)	1/55 (1.8%)	1/93 (1.1%)	p > .99*
Alcoholism	2/38 (5.3%)	4/55 (7.3%)	6/93 (6.5%)	p > .99*
Tobacco consumption	9/38 (23.7%)	15/55 (27.3%)	24/93 (25.8%)	p = .81*
Malignancy	4/38 (10.5%)	3/55 (5.5%)	7/93 (7.5%)	p = .44*
Psychiatric condition	2/38 (5.3%)	3/55 (5.5%)	5/93 (5.4%)	p > .99*
Neurologic/neurosurgical condition	3/38 (7.9%)	4/55 (7.3%)	7/93 (7.5%)	p > .99*
Cardiovascular condition	2/38 (5.3%)	3/55 (5.5%)	5/93 (5.4%)	p > .99*
Pulmonary condition	4/38 (10.5%)	7/55 (12.7%)	11/93 (11.8%)	p > .99*
Rheumatic condition	3/38 (7.9%)	0/55 (0.0%)	3/93 (3.2%)	p = .07*
Cardiopulmonary resuscitation	0/38 (0.0%)	3/55 (5.5%)	3/93 (3.2%)	p = .27*
Rebleeding	3/38 (7.9%)	6/55 (10.9%)	9/93 (9.7%)	p = .73*
Delayed cerebral ischemia	14/38 (36.8%)	22/55 (40.0%)	36/93 (38.7%)	p = .83*
Seizure	7/38 (18.4%)	5/55 (9.1%)	12/93 (12.9%)	p = .22*

*Fisher’s exact test

### Headache description, pain-related restrictions, and accompanying complaints

A total of 40.9% (38/93) of all investigated good-grade SAH patients were affected by burdensome headache. In total, 47% of the 38 HA+ patients suffered from pain attacks free of continuing background headache, whereas 53% indicated persistent cephalalgia with or without additional pain attacks. A total of 23% of HA+ cases had one or more headache attacks per day, and half of HA+ patients reported on diurnal patterns in respect of the headache. The subjective perception of headache by means of the pain description list of the DSF can be obtained from Table 4. The Mean average and maximum NRS pain scores of the HA+ group during the last 4 weeks were 3.7 ± 2.3 and 5.7 ± 2.9 which is significantly higher than in the HA- group (0.5 ± 1.3 average pain; 0.5 ± 1.5 maximum pain; both *p* < .01). Mean SF-MPQ total pain index was significantly different between HA+ (10.7 ± 8.3) and HA- cases (0.7 ± 2.9; p < .01). Regular intake of non-opioid and opioid analgesics was more commonly seen in HA+ (42%) than in HA- patients (7%; *p* < .01). Correspondingly, HDI-G emotional and functional scores of the HA+ cohort indicated markedly higher values compared to the HA- group representing more pronounced degrees of limitation (emotional score: 17.7 ± 14.6 HA+ versus 0.4 ± 1.3 HA-; functional score: 17.4 ± 12.9 HA+ versus 0.9 ± 3.0 HA-; both *p* < .01). The HA+ patients complained about an average number of 9.2 ± 18.0 days with considerable pain-related restrictions of daily living activities **(ADL)** during the last 3 months, while the HA- cohort was almost unaffected (0.7 ± 3.4 days; p < .01). Besides the ADL, also recreational and working activities as well as general mood were significantly impaired in HA+ patients, as shown in addition to other relevant pain-related measures in Table 5. Eventually, HA+ patients realized numerous further complaints such as listlessness, weariness, and concentration problems far more frequently than HA- patients. Table 6 contrasts both groups with regard to the prevalence of concomitant symptoms derived from the add-on module A of the DSF.

**Table 4:** Self-reported features of burdensome headache. *Affective scale (range 0–12 with 12 points indicating maximum affective pain experience) of the pain description list of the German Pain Questionnaire

Dull	11/38 (28.9%)
Pressing	19/38 (50.0%)
Throbbing	9/38 (23.7%)
Knocking	5/38 (13.2%)
Stabbing	18/38 (47.4%)
Pulling	14/38 (36.8%)
Hot	3/38 (7.9%)
Burning	7/38 (18.4%)
Mean affective scale*/SD	3.0/3.3

**Table 5:** Specification of burdensome headache in SAH patients.* Mann-Whitney test

	SAH with headache	SAH without headache	Significance	Comment
Type of headache_continuous without attacks	12/38 (31.6%)	Not applicable	Not applicable	Group a
Type of headache_continuous with attacks	8/38 (21.1%)	Not applicable	Not applicable	Group b
Type of headache_only pain attacks	18/38 (47.4%)	Not applicable	Not applicable	Group c
Frequency of headache attacks_daily or more	6/26 (23.1%)	Not applicable	Not applicable	Only patients with pain attacks (group b + c) evaluated
Frequency of headache attacks_weekly to almost daily	7/26 (26.9%)	Not applicable	Not applicable	Only patients with pain attacks (group b + c) evaluated
Diurnal rhythm of headache	18/34 (52.9%)	Not applicable	Not applicable	
Mean current pain (NRS)/SD	2.1/1.8	0.3/0.9	p < .01*	
Mean average pain (NRS)/SD	3.7/2.3	0.5/1.3	p < .01*	Last 4 weeks
Mean maximum pain (NRS)/SD	5.7/2.9	0.5/1.5	p < .01*	Last 4 weeks
Mean number of days with pain-related limitation of ADL/SD	9.2/18.0	0.7/3.4	p < .01*	Last 3 months
Mean pain-related limitation of ADL (NRS)/SD	3.0/3.1	0.3/1.0	p < .01*	Last 3 months
Mean pain-related limitation of recreational activities (NRS)/SD	4.1/3.4	0.3/1.0	p < .01*	Last 3 months
Mean pain-related limitation of working activities (NRS)/SD	4.4/3.7	0.3/1.1	p < .01*	Last 3 months
Pain-related impairment of mood	1/1–2	0/0–0	p < .01*	0 = no; I = slight; II = significant; III = much; IV = very much
Mean HDI-G_emotional score/SD	17.7/14.6	0.4/1.3	p < .01*	
Mean HDI-G_functional score/SD	17.4/12.9	0.9/3.0	p < .01*	
Median HDI-G_headache days/25%–75% interval	1/0–2	0/0–0	p < .01*	Days per month: 0 = <1 day; 1 = 1–4 days; 2= > 4 days
Mean SF-MPQ_total pain index/SD	10.7/8.3	0.7/2.9	p < .01*	
Median SF-MPQ_pain experience/25%–75% interval	2/1–4	0/0–0	p < .01*	
Pain medication	0/0–1	0/0–0	p < .01*	0 = no analgesics; I = non-opioids; 2 = opioids ± non-opioids

ADL = activities of daily living, HDI-G = German version of the Henry Ford Hospital Headache Disability Inventory, NRS = numeric rating scale (range 0–10), SD = standard deviation, SF-MPQ = short-form McGill Pain Questionnaire

**Table 6:** Concomitant symptoms of SAH patients with and without burdensome headache

Symptom	SAH with headache	SAH without headache	SAH total	Significance
No other symptoms	6/38 (15.8%)	33/55 (60.0%)	39/93 (41.9%)	p< .01*
Weariness	25/38 (65.8%)	8/55 (14.5%)	33/93 (35.5%)	p < .01*
Nausea	8/38 (21.1%)	2/55 (3.6%)	10/93 (10.8%)	p = .01*
Stomach complaints	6/38 (15.8%)	2/55 (3.6%)	8/93 (8.6%)	p = .06*
Concentration problems	18/38 (47.4%)	13/55 (23.6%)	31/93 (33.3%)	p = .02*
Dejection	14/38 (36.8%)	9/55 (16.4%)	23/93 (24.7%)	p = .03*
Lack of appetite	7/38 (18.4%)	2/55 (3.6%)	9/93 (9.7%)	p = .03*
Sweating	12/38 (31.6%)	2/55 (3.6%)	14/93 (15.1%)	p < .01*
Listlessness	19/38 (50.0%)	12/55 (21.8%)	31/93 (33.3%)	p = .01*
Dizziness	17/38 (44.7%)	10/55 (18.2%)	27/93 (29.0%)	p = .01*
Obstipation	4/38 (10.5%)	3/55 (5.5%)	7/93 (7.5%)	p = .44*

*Fisher’s exact test

### Health-related quality of life, psychosocial, and functional impairment

HA+ patients showed reduced HR-QOL measures in comparison to HA- cases at follow-up. While mean SF-12 physical composite scores were pronouncedly lower in the HA+ group (40.3 ± 9.9 versus 49.6 ± 8.6; *p* < .01), substantial significant differences between both cohorts could not be determined with respect to SF-12 mental composite scores (44.0 ± 11.8 HA + versus 49.2 ± 9.6 HA-; *p* = .06). Depression, anxiety, and stress levels evaluated by means of the DASS were increased in HA+ in relation to HA- cases. Results were significant for the anxiety (4.0 ± 4.4 versus 2.0 ± 2.6; *p* = .02) and stress subscores (8.3 ± 6.1 versus 4.9 ± 4.9; *p* = .01). In accordance with the described mental tension, 41.7% of the HA+ patients suffered from insufficient night sleep, whereas only 14.6% of the HA- collective reported on sleep disturbances (p = .01). A total of 49.3% of SAH cases (GOS ≥ 3) were recognized by the pension offices as having a degree of disability, and 29.2% of SAH patients became incapacitated for work at follow-up without significant differences in view of headache occurrence. All group comparisons regarding quality of life, psychosocial, and functional interferences are set out in detail in Table 7.

**Table 7:** Health-related quality of life, psychosocial wellbeing, and functional capacity of SAH patients with and without burdensome headache

	SAH with headache	SAH without headache	SAH total	Significance
Mean general wellbeing*/SD	15.1/59.8	32.1/55.1	24.0/57.6	p = .19***
Mean SF-12 PCS/SD	40.3/9.9	49.6/8.6	45.0/10.3	p < .01***
Mean SF-12 MCS/SD	44.0/11.8	49.2/9.6	46.7/11.0	p = .06***
Mean DASS_depression/SD	6.0/6.1	3.9/4.9	5.0/5.6	p = .10***
Mean DASS_anxiety/SD	4.0/4.4	2.0/2.6	3.0/3.7	p = .02***
Mean DASS_stress/SD	8.3/6.1	4.9/4.9	6.6/5.8	p = .01***
Emotional/psychic problems	14/38 (36.8%)	11/41 (26.8%)	25/79 (31.6%)	p = .47****
Insufficient night sleep	15/36 (41.7%)	6/41 (14.6%)	21/77 (27.3%)	p = .01****
Median social impairment**/25%–75% interval	2/1–2	1/0–2	1/0–2	p = .01***
Officially certified physical disability	20/36 (55.6%)	16/37 (43.2%)	36/73 (49.3%)	p = .35****
Incapacity for work	11/32 (34.4%)	8/33 (24.2%)	19/65 (29.2%)	p = .42****

*Module A of the German pain questionnaire (range: −100 to +100), **Question 12 of the 12-Item Short-Form Health Survey (0 = never; 1 = rarely; 2 = sometimes; 3 = mostly; 4 = always), *** Mann-Whitney test or unpaired t-test, **** Fisher’s exact test, DASS = Depression Anxiety and Stress Scale, SD = standard deviation, SF-12 MCS = Mental Health Composite Score of the 12-Item Short-Form Health Survey, SF-12 PCS=Physical Health Composite Score of the 12-Item Short-Form Health Survey

### Predictability of burdensome headache following SAH

Multivariate logistic regression of parameters with significant group differences revealed age at bleeding onset (OR for each added year 0.945; CI 0.903–0.989; *p* = .01) and initial WFNS grade (OR for each degree of progression 0.577; CI 0.335–0.994; *p* = .048) as the only factors which were independently associated with headache in the long run following SAH. GOS scores at hospital discharge demonstrated no significant predictive value.

## Discussion

Data on the incidence and impact of chronic headache after SAH is sparse. Here, we demonstrate that a significant number of the SAH patients suffer from burdensome headaches with a profound negative impact on the HR-QOL. Our SAH study collective presented with a mean age of 52.5 years and two out of three cases were of female gender. Previous studies on SAH reported on average patient ages between 50 and 57 years with males in general being affected earlier [7, 39, 66, 69]. Throughout the body of literature, females suffer more frequently from SAH than their male counterparts after their late thirties [39, 69]. With respect to the above-mentioned preceding investigations, our patient sample is in line with the main epidemiological SAH characteristics and, therefore, representative. A total of 78.5% of our patients suffered from bleeding as a result of an aneurysm located at the anterior cerebral circulation with a mean aneurysm size of 5.9 ± 3.2 mm, which is in line with other studies [2, 19, 28, 69]. The SAH severity distribution of the study population differed significantly from other surveys which generally give account of lower percentages of patients grouped into neurologically favorable low SAH grades [2, 39]. The higher percentage of lower scores determined in this study may be explained by the predefined inclusion criterion of a GOS ≥ 3 at discharge, because a positive correlation between advantageous neurological condition at hospital admission and good functional outcome has been proven for patients suffering from SAH [15]. In our series a minority of patients (28%) underwent microsurgical clipping. Historically, the major share of SAH cases were allocated to open surgical procedures [39], but over the past decades, the ratio of patients treated with endovascular devices (e.g., coiling embolization) grew continuously forming nowadays the treatment of choice for the majority of cases [37, 59]. Our results suggest that long-term prevalence of headache does not seem to be dependent on the chosen treatment modality with similar coiling-clipping quota in both groups (HA+ and HA-). This conclusion contradicts the findings of a recent study reporting lower pain scores after open surgical intervention at 6 months follow-up [10]. Of note, apart from a divergent follow-up period and a small patient sample, this study refers to pain in general and is therefore not headache-specific. Younger SAH patients were more likely to develop headache at follow-up, which is in conformity with the findings of a recently published meta-analysis suggesting younger patients to be more prone to suffer from headache following ischemic stroke [25]. A possible reason for the higher prevalence of burdensome headache in SAH patients without major deficits (WFNS I/II) could be the fact that these patients generally are aware of the potentially traumatizing treatment course while being in intensive care. Roper and colleagues found a correlation between post-traumatic stress disorder and headache-related limitations following traumatic brain injury [58]. Preexisting comorbidities and early in-hospital complications such as rebleeding or delayed cerebral ischemia were not significantly different between patients with and without headache. Our SAH cases showed equivalent cardiopulmonary comorbidity rates as well as similar ratios for alcohol and tobacco consumption compared to previous studies, but diabetes mellitus was less frequently encountered in our cohort [2, 39]. Interestingly, all three SAH patients with anamnestic prior rheumatic condition reported on burdensome headache at follow-up, but this association narrowly failed the statistical significance. Being described already in 1938 by Cyriax, many recent reports chronicle headache in conjunction with various rheumatic conditions like polymyalgia rheumatica, systemic lupus erythematosus, and antiphospholipid syndrome [11, 14, 53, 64]. Conversely, people with headache had a higher prevalence of rheumatic disorders in an Austrian general population-based study [55]. Cavestro et al. gave additional insight into the linkage between headache and various systemic autoimmune diseases [13]. Fatigue, a common feature in rheumatic disorders, could be a factor contributing to the occurrence of chronic headache in this setting and has already been recognized as a risk factor for persistent headache after ischemic stroke [38]. The HA+ group comprising 40.9% (38/93) of good-grade SAH patients suffered from an average headache intensity of 3.7/10 NRS at long-term follow-up, which came along with approximately 9 days of significant restrictions of daily living activities within the last 3 months. According to other empirical surveys, 77% of good-grade SAH patients and 16–22% of SAH long-term survivors presented with higher headache burden several years after the bleeding event [12, 30, 51]. A Danish study reported on persistent primarily tension-type or migraine-like headache 3 years after stroke in about 10% of patients with a comparable mean intensity of 4.5/10 NRS during the last weeks [24]. The body of evidence suggests SAH to be a subtype of stroke with especially high propensity toward headache in the acute and chronic setting [22]. Even though episodic tension-type headache and migraine are known to show a high lifetime prevalence [52], a relatively low headache point prevalence of 5.7% was stated in a Central European population-based study [55]. Hence, persistent headache seems to be much more prevalent in SAH survivors compared to the general population, which also contributes to elevated health-related expenditures mainly by means of indirect costs [17]. Interestingly, one recent case-control study revealed an increased migraine frequency also in patients with unruptured intracranial aneurysms [68]. The pathophysiology behind the development of persistent post-stroke headache is principally not understood so far and thus speculative. One possible mode of action could be the central sensitization of nociceptive pathways [38]. Another possible mechanism could be chronic hyperreactivity of the cerebral vasculature, as it is also seen in the so-called reversible cerebral vasoconstriction syndrome, which is typically characterized by chronic headache with intermittent thunderclap attacks [46]. Headache tends to be more frequent after cortical ischemic strokes as compared to subcortical events. Moreover, headache is more likely present following a large vessel than small vessel occlusion [25]. The subarachnoid space, which is the region of interest in SAH, is directly adjacent to the cortex. Therefore, ischemic and hemorrhagic vascular events may affect the same anatomical cortical structures by means of different mechanisms (ischemia, aseptic inflammation, delayed cerebral ischemia). Currently, there is a lack of knowledge with regard to the pathophysiological key processes leading to post-SAH headache preventing specific target-oriented treatment measures. Generally speaking, an essential step in chronic headache management besides analgesic medication is the increase in patient resiliency. This can be primarily achieved by cognitive-behavioral approaches and relaxation training [61]. A multidisciplinary approach including physiotherapy seems to be the most preferable therapeutic strategy [20]. We found long-lasting headache after SAH in association with pronounced weariness, sleep disturbance, and concentration problems. Numerous authors reported on high rates of chronic tiredness, motivational impairment, sleep disorder, and various cognitive limitations in patients who were treated for SAH beforehand [3, 30, 40, 51, 56], but there are also contradictory findings suggesting sufficient neuropsychological recovery following hemorrhage [36, 54]. In line with our findings, a recent comprehensive literature review also identified reduced well-being related to diminished energy level as well as sleep impairment in patients with primary headache disorders [1]. The etiology of reduced vitality following SAH has not been fully elucidated yet, but development of hypothyroidism could be a contributing factor with partial recovery of cognitive dysfunction after hormone replacement therapy [42]. In our study, HR-QOL was significantly decreased in HA+ patients along with increased levels of anxiety and stress indicating a profound impact of burdensome headache on patient’s quality of life perception. Despite a general lack of neuropsychological and QOL endpoints in most interventional SAH trials [5], there is a growing body of evidence regarding anxiety, stress, and depression as frequently encountered phenomena in long-term SAH survivors, which may subsequently contribute to reduced HR-QOL and social isolation [30, 34, 36, 56]. The HR-QOL restrictions in SAH patients seem to be stable over at least two years post hemorrhage [65]. Bodily, social, and role functioning limitations are well-known factors undermining HR-QOL in patients suffering from any type of chronic headache [1, 8]. One out of two of the whole SAH sample in this study presented with an officially certified degree of disability, and almost one out of three patients were incapacitated for work at follow-up which states the enormous socioeconomic importance of this certain subtype of stroke affecting preferentially people of younger ages. Our findings concerning long-term disability and occupational reintegration difficulties agree with the current state of scientific knowledge [36, 51, 60, 70]. The HA+ patients revealed slightly higher, but non-significant levels of disability and unemployability. Monzani et al. determined a correlation between headache intensity and loss of workers’ productivity in patients suffering from tension-type headache [45]. A recent comprehensive meta-ethnographic systematic analysis describes chronic headache as “driver of behavior’ which subsequently provokes a feeling of “a loss of control” in patient’s life. This loss of control significantly contributes to the self-experienced limitations [48]. For migraineurs it has been shown that these patients are less physically active and mobile than controls even in their interictal (pain-free) phases [62]. This finding underlines an intensity-independent disabling effect of headache conditions. The main strengths of this investigation are the prospective data collection of a large and up-to-date consecutive patient sample (*n* = 93) from an extensive catchment area, a long follow-up period (mean 32.6 months, range between 14 and 50 months), and the utilization of a comprehensive multidimensional set of validated pain and physical as well as mental health measures. Standardized pain questionnaires are not designed to establish a specific headache diagnosis, and therefore, we are incapable of making clear statements regarding the distribution of different headache entities with respect to the International Classification of Headache Disorders of the International Headache Society. We are aware of the potential bias due to the refusal to participate of a fraction of patients and the per-protocol exclusion of poor-grade SAH patients. Another possible limitation, which should be mentioned here, is the monocentric study design.

## Conclusion

Our results suggest that a considerable proportion of good-grade SAH patients suffers from sustained burdensome headache at long-term follow-up with significant pain-related impairment of everyday activities. Furthermore, persistent headache seems to be associated with increased weariness, sleep disturbance, cognitive dysfunction, as well as anxiety and stress levels, which may altogether contribute to a relevant reduction in the quality of life in SAH cases. Consequently, there is an urgent need for further basic and clinical research in this field to elucidate the underlying pathophysiological and psychophysiological mechanisms, characterize the specific phenotype of long-term post-SAH headache, and also develop preventive as well as therapeutic pain reduction strategies following SAH.

## Electronic supplementary material

ESM 1 (DOCX 27.9 kb)

## Abbreviations

ADL: activities of daily living
CI: 95% confidence interval
DASS: Depression, Anxiety and Stress scale
DSF: German pain questionnaire of the German Society for the Study of Pain
GOS: Glasgow Outcome Scale
HA±: subarachnoid hemorrhage patients with (HA+) or without (HA-) burdensome headache
HDI-G: German version of the Henry Ford Hospital Headache Disability Inventory
HR-QOL: health-related quality of life
ICD-10-GM: German modification of the 10th revision of the International Statistical Classification of Diseases and Related Health Problems
ICU: intensive care unit
NRS: 10-point numeric pain rating scale
OR: odds ratio
SAH: aneurysmal subarachnoid hemorrhage
SD: standard deviation
SF-12: 12-Item Short-Form Health Survey
SF-MPQ: short-form McGill Pain Questionnaire
WFNS: World Federation of Neurological Surgeons Subarachnoid Hemorrhage Grading

## References

[1] Abu Bakar N, Tanprawate S, Lambru G, Torkamani M, Jahanshahi M, Matharu M (2016). Quality of life in primary headache disorders: a review. Cephalalgia.

[2] Abulhasan YB, Alabdulraheem N, Simoneau G, Angle MR, Teitelbaum J (2018). Mortality after spontaneous subarachnoid hemorrhage: causality and validation of a prediction model. World Neurosurg.

[3] Al-Khindi T, Macdonald RL, Schweizer TA (2010) Cognitive and functional outcome after aneurysmal subarachnoid hemorrhage. Stroke. 10.1161/STROKEAHA.110.581975 10.1161/STROKEAHA.110.581975 20595669

[4] Alleyne CH (2010). Aneurysmal subarachnoid hemorrhage: have outcomes really improved?. Neurology.

[5] Andersen CR, Fitzgerald E, Delaney A, Finfer S (2019). A systematic review of outcome measures employed in aneurysmal subarachnoid hemorrhage (aSAH) clinical research. Neurocrit Care.

[6] Bauer B, Evers S, Gralow I, Husstedt IW (1999). Psychosocial handicap due to chronic headaches. Evaluation of the inventory of disabilities caused by headache. Nervenarzt.

[7] Bederson JB, Connolly ES, Batjer HH, Dacey RG, Dion JE, Diringer MN, Duldner JE, Harbaugh RE, Patel AB, Rosenwasser RH (2009). Guidelines for the management of aneurysmal subarachnoid hemorrhage: a statement for healthcare professionals from a special writing group of the Stroke Council, American Heart Association. Stroke.

[8] Bera S, Goyal V, Khandelwal S, Sood M (2014). A comparative study of psychiatric comorbidity, quality of life and disability in patients with migraine and tension type headache. Neurol India.

[9] Broderick JP, Brott TG, Duldner JE, Tomsick T, Leach A (1994). Initial and recurrent bleeding are the major causes of death following subarachnoid hemorrhage. Stroke.

[10] Bründl E, Schödel P, Bele S, Proescholdt M, Scheitzach J, Zeman F, Brawanski A, Schebesch K-M (2018). Treatment of spontaneous subarachnoid hemorrhage and self-reported neuropsychological performance at 6 months - results of a prospective clinical pilot study on good-grade patients. Turk Neurosurg.

[11] Buttgereit F, Dejaco C, Matteson EL, Dasgupta B (2016). Polymyalgia rheumatica and giant cell arteritis: a systematic review. JAMA.

[12] Canhao P, Ferro JM, Pinto AN, Melo TP, Campos JG (1995). Perimesencephalic and nonperimesencephalic subarachnoid haemorrhages with negative angiograms. Acta Neurochir.

[13] Cavestro C, Ferrero M (2018). Migraine in systemic autoimmune diseases. Endocr Metab Immune Disord - Drug Targets.

[14] Cyriax J (1938). Rheumatic headache. Br Med J.

[15] D’Souza S (2015) Aneurysmal subarachnoid hemorrhage. J Neurosurg Anesthesiol 27(3):222–240 10.1097/ANA.0000000000000130 PMC4463029 25272066

[16] Etminan N, Chang H-S, Hackenberg K, de Rooij NK, Vergouwen MDI, Rinkel GJE, Algra A (2019). Worldwide incidence of aneurysmal subarachnoid hemorrhage according to region, time period, blood pressure, and smoking prevalence in the population: a systematic review and meta-analysis. JAMA Neurol.

[17] Evers S, Frese A, Marziniak M (2006). Differential diagnosis of headache. Dtsch Aerzteblatt Online.

[18] Fisher CM, Kistler JP, Davis JM (1980). Relation of cerebral vasospasm to subarachnoid hemorrhage visualized by computerized tomographic scanning. Neurosurgery.

[19] Froelich JJ, Neilson S, Peters-Wilke J, Dubey A, Thani N, Erasmus A, Carr MW, Hunn AWM (2016). Size and location of ruptured intracranial aneurysms: a 5-year clinical survey. World Neurosurg.

[20] Gaul C, Liesering-Latta E, Schäfer B, Fritsche G, Holle D (2016). Integrated multidisciplinary care of headache disorders: a narrative review. Cephalalgia Int J Headache.

[21] German Pain Society German chapter of the International Association for the Study of Pain. https://www.dgss.org/startseite/. Accessed 7 Jul 2019

[22] Gorelick PB, Hier DB, Caplan LR, Langenberg P (1986). Headache in acute cerebrovascular disease. Neurology.

[23] Graeb DA, Robertson WD, Lapointe JS, Nugent RA, Harrison PB (1982). Computed tomographic diagnosis of intraventricular hemorrhage. Etiology and prognosis. Radiology.

[24] Hansen AP, Marcussen NS, Klit H, Kasch H, Jensen TS, Finnerup NB (2015). Development of persistent headache following stroke: a 3-year follow-up. Cephalalgia.

[25] Harriott AM, Karakaya F, Ayata C (2019) Headache after ischemic stroke: a systematic review and meta-analysis. Neurology. 10.1212/WNL.0000000000008591 10.1212/WNL.0000000000008591 PMC7011689 31694924

[26] Headache Classification Subcommittee of the International Headache Society (2004). The international classification of headache disorders: 2nd edition. Cephalalgia Int J Headache.

[27] Hijdra A, Brouwers PJ, Vermeulen M, van Gijn J (1990). Grading the amount of blood on computed tomograms after subarachnoid hemorrhage. Stroke.

[28] Hostettler IC, Alg VS, Shahi N (2018). Characteristics of Unruptured compared to ruptured intracranial aneurysms: a multicenter case–control study. Neurosurgery.

[29] Hughes JD, Bond KM, Mekary RA, Dewan MC, Rattani A, Baticulon R, Kato Y, Azevedo-Filho H, Morcos JJ, Park KB (2018). Estimating the global incidence of aneurysmal subarachnoid hemorrhage: a systematic review for central nervous system vascular lesions and meta-analysis of ruptured aneurysms. World Neurosurg.

[30] Hütter BO, Gilsbach JM, Kreitschmann I (1995). Quality of life and cognitive deficits after subarachnoid haemorrhage. Br J Neurosurg.

[31] Ikawa F, Ohbayashi N, Imada Y, Matsushige T, Kajihara Y, Inagawa T, Kobayashi S (2004). Analysis of subarachnoid hemorrhage according to the Japanese standard stroke registry study-incidence, outcome, and comparison with the international subarachnoid aneurysm trial. Neurol Med Chir (Tokyo).

[32] Jacobson GP, Ramadan NM, Aggarwal SK, Newman CW (1994). The Henry ford hospital headache disability inventory (HDI). Neurology.

[33] Jennett B, Bond M (1975). Assessment of outcome after severe brain damage. Lancet Lond Engl.

[34] Katati MJ, Santiago-Ramajo S, Pérez-García M, Meersmans-Sánchez Jofré M, Vilar-Lopez R, Coín-Mejias MA, Caracuel-Romero A, Arjona-Moron V (2007). Description of quality of life and its predictors in patients with aneurysmal subarachnoid hemorrhage. Cerebrovasc Dis.

[35] Kelley K (2003). Good practice in the conduct and reporting of survey research. Int J Qual Health Care.

[36] Krajewski K, Dombek S, Martens T, Köppen J, Westphal M, Regelsberger J (2014). Neuropsychological assessments in patients with aneurysmal subarachnoid hemorrhage, perimesencephalic SAH, and incidental aneurysms. Neurosurg Rev.

[37] La Pira B, Singh TD, Rabinstein AA, Lanzino G (2018). Time trends in outcomes after aneurysmal subarachnoid hemorrhage over the past 30 years. Mayo Clin Proc.

[38] Lai J, Harrison RA, Plecash A, Field TS (2018). A narrative review of persistent post-stroke headache - a new entry in the international classification of headache disorders, 3rd edition. Headache.

[39] Lantigua H, Ortega-Gutierrez S, Schmidt JM, Lee K, Badjatia N, Agarwal S, Claassen J, Connolly ES, Mayer SA (2015). Subarachnoid hemorrhage: who dies, and why?. Crit Care.

[40] Ljunggren B, Sonesson B, Säveland H, Brandt L (1985). Cognitive impairment and adjustment in patients without neurological deficits after aneurysmal SAH and early operation. J Neurosurg.

[41] Lovelock CE, Rinkel GJE, Rothwell PM (2010). Time trends in outcome of subarachnoid hemorrhage: population-based study and systematic review. Neurology.

[42] Ma J, Yang X, Yin H, Wang Y, Chen H, Liu C, Han G, Gao F (2015). Effect of thyroid hormone replacement therapy on cognition in long-term survivors of aneurysmal subarachnoid hemorrhage. Exp Ther Med.

[43] Mayberg MR, Batjer HH, Dacey R, Diringer M, Haley EC, Heros RC, Sternau LL, Torner J, Adams HP, Feinberg W (1994). Guidelines for the management of aneurysmal subarachnoid hemorrhage. A statement for healthcare professionals from a special writing group of the stroke council, American Heart Association. Stroke.

[44] Melzack R (1987). The short-form McGill pain questionnaire. Pain.

[45] Monzani L, Zurriaga R, Espí López GV (2018). Anxiety and the severity of tension-type headache mediate the relation between headache presenteeism and workers’ productivity. PLoS One.

[46] Muehlschlegel S, Kursun O, Topcuoglu MA, Fok J, Singhal AB (2013) Differentiating reversible cerebral vasoconstriction syndrome with subarachnoid hemorrhage from other causes of subarachnoid hemorrhage. JAMA Neurol. 10.1001/jamaneurol.2013.3484 10.1001/jamaneurol.2013.3484 23939614

[47] Nagel B, Gerbershagen HU, Lindena G, Pfingsten M (2002). Development and evaluation of the multidimensional German pain questionnaire. Schmerz Berl Ger.

[48] Nichols VP, Ellard DR, Griffiths FE, Kamal A, Underwood M, Taylor SJC (2017). The lived experience of chronic headache: a systematic review and synthesis of the qualitative literature. BMJ Open.

[49] Nilges P, Essau C (2015). Depression, anxiety and stress scales: DASS--A screening procedure not only for pain patients. Schmerz Berl Ger.

[50] No authors (1988) Report of world Federation of Neurological Surgeons Committee on a universal subarachnoid hemorrhage grading scale. J Neurosurg 68(6):985–986 10.3171/jns.1988.68.6.0985 3131498

[51] Ogden JA, Utley T, Mee EW (1997). Neurological and psychosocial outcome 4 to 7 years after subarachnoid hemorrhage. Neurosurgery.

[52] Rasmussen BK (1995). Epidemiology of headache. Cephalalgia Int J Headache.

[53] Ricarte IF, Dutra LA, Abrantes FF, Toso FF, Barsottini OGP, Silva GS, de Souza AWS, Andrade D (2018). Neurologic manifestations of antiphospholipid syndrome. Lupus.

[54] Richardson JTE (1991). Cognitive performance following rupture and repair of intracranial aneurysm. Acta Neurol Scand.

[55] Rieder A, Lobentanz I, Zeitlhofer J, Mitsche N, Lawrence K, Schwarz B, Kunze M (2004). Background morbidity of headache in an adult general population. Results of the Austrian SERMO (self-reported morbidity) study. Wien Klin Wochenschr.

[56] Rinkel GJ, Algra A (2011). Long-term outcomes of patients with aneurysmal subarachnoid haemorrhage. Lancet Neurol.

[57] de Rooij NK, Linn FHH, van der Plas JA, Algra A, Rinkel GJE (2007). Incidence of subarachnoid haemorrhage: a systematic review with emphasis on region, age, gender and time trends. J Neurol Neurosurg Psychiatry.

[58] Roper LS, Nightingale P, Su Z, Mitchell JL, Belli A, Sinclair AJ (2017). Disability from posttraumatic headache is compounded by coexisting posttraumatic stress disorder. J Pain Res.

[59] Smith GA, Dagostino P, Maltenfort MG, Dumont AS, Ratliff JK (2011). Geographic variation and regional trends in adoption of endovascular techniques for cerebral aneurysms. J Neurosurg.

[60] Sonesson B, Kronvall E, Säveland H, Brandt L, Nilsson OG (2018). Long-term reintegration and quality of life in patients with subarachnoid hemorrhage and a good neurological outcome: findings after more than 20 years. J Neurosurg.

[61] Stonnington CM, Kothari DJ, Davis MC (2016). Understanding and promoting resiliency in patients with chronic headache. Curr Neurol Neurosci Rep.

[62] Stronks DL, Tulen JHM, Bussmann JBJ, Mulder LJMM, Passchier J (2004). Interictal daily functioning in migraine. Cephalalgia Int J Headache.

[63] Teasdale G, Jennett B (1974). Assessment of coma and impaired consciousness. A practical scale. Lancet Lond Engl.

[64] Torrente-Segarra V, Salman Monte TC, Rúa-Figueroa I (2017). Juvenile- and adult-onset systemic lupus erythematosus: a comparative study in a large cohort from the Spanish Society of Rheumatology Lupus Registry (RELESSER). Clin Exp Rheumatol.

[65] von Vogelsang A-C, Thelin EP, Hakim R, Svensson M (2017) Health-related quality of life dynamics 2 years following aneurysmal subarachnoid hemorrhage: a prospective cohort study using EQ-5D. Neurosurgery. 10.1093/neuros/nyx059 10.1093/neuros/nyx059 28368438

[66] von Vogelsang A-C, Wengström Y, Svensson M, Forsberg C (2013) Descriptive epidemiology in relation to gender differences and treatment modalities 10 years after intracranial aneurysm rupture in the Stockholm cohort 1996–1999. World Neurosurg 80(3–4):328–334 10.1016/j.wneu.2012.06.041 22898030

[67] Ware J, Kosinski M, Keller SD (1996). A 12-item short-form health survey: construction of scales and preliminary tests of reliability and validity. Med Care.

[68] Witvoet EH, Pelzer N, Terwindt GM, Rinkel GJE, Vlak MHM, Algra A, Wermer MJH (2017). Migraine prevalence in patients with unruptured intracranial aneurysms: a case-control study. Brain Behav.

[69] Zumofen DW, Roethlisberger M, Achermann R (2018). Factors associated with clinical and radiological status on admission in patients with aneurysmal subarachnoid hemorrhage. Neurosurg Rev.

[70] Zweifel-Zehnder AE, Stienen MN, Chicherio C (2015). Call for uniform neuropsychological assessment after aneurysmal subarachnoid hemorrhage: Swiss recommendations. Acta Neurochir.

