# Supplementary material for: The burden of headache following aneurysmal subarachnoid hemorrhage: a prospective single-center cross-sectional analysis
Source: Acta Neurochir (Wien). 2020 Feb 4;162(4):893–903. doi: 10.1007/s00701-020-04235-7 (PMC7066282; doi:10.1007/s00701-020-04235-7)
Supplement: Supplementary file 1 — (DOCX 27.9 kb) [file 701_2020_4235_MOESM1_ESM.docx]

**Key points regarding the principles of good practice in the reporting of survey research**

The checklist was adopted from the general considerations concerning good practice in the conduct and reporting of survey research proposed by Kelley et al. in 2003.^1^

1. **Explain the purpose or aim of the research, with the explicit identification of the research question.**

*As outlined in the `background and objective` section, this study aims to characterize the long-term burden of headache and its influence on health-related quality of life after aneurysmal subarachnoid hemorrhage.*

1. **Explain why the research was necessary and place the study in context, drawing upon previous work in relevant fields.**

*By now, the main body of SAH literature lacks a detailed coverage of patient reported outcome measures. The long-term burden of chronic headache and its association with health-related quality of life limitations has not been sufficiently elucidated so far.*

1. **Describe in detail how the research was done.**

**State the chosen research method or methods, and justify why this method was chosen.**

*An appropriate instrument for the collection of patient reported measures like burdensome long-term headache is a standardized interview, which was performed prospectively by phone and subsequent postal survey*.

**Describe the research tool. If an existing tool is used, briefly state its psychometric properties and provide references to the original development work. If a new tool is used, you should include an entire section describing the steps undertaken to develop and test the tool, including results of psychometric testing.**

*We used validated pain and health-related quality of life questionnaires with well-established psychometric properties:*

*-German Pain Questionnaire (DSF)*^2^

*-German version of the Henry Ford Hospital Headache Disability Inventory (HDI-G)*^3^

*-Short-Form McGill Pain Questionnaire (SF-MPQ)*^4^

*-12-Item Short-Form Health Survey (SF-12)*^5^

*-Depression, Anxiety and Stress scale (DASS)*^6^

**Describe how the sample was selected and how data were collected.**

*The investigation comprised all aneurysmal SAH patients treated at our institution between 1/1/2014 and 12/31/2016 with good neurological status at hospital discharge (Glasgow Outcome Scale ≥ 3). Data were collected prospectively.*

**How were potential subjects identified?**

*Subjects were identified by thorough medical chart review.*

**How many and what type of attempts were made to contact subjects?**

*At first, subjects were contacted via phone call (study information, informed consent). At least 3 attempts per patient were performed on different days. In case of missing patient contact information we got in touch with the corresponding family physician to obtain current phone and address data. Secondly, a postal survey was sent to patients willing to participate in the study.*

**Who approached potential subjects?**

*All subjects were approached by the principal investigator/corresponding author*.

**Where were potential subjects approached?**

*Potential subjects were approached at home (phone call and postal survey).*

**How was informed consent obtained?**

*Subjects were informed about the purpose of the study as well as expected time expense in case of participation via phone conversation. An informed consent form was sent to the patients willing to take part in the survey together with the set of questionnaires. Patients were included in the analysis only if written informed consent was returned to our institution.*

**How many agreed to participate?**

*93 out of 145 patients meeting the eligibility criteria agreed to participate. Main reasons for non-participation were unknown new address (26X), rejection (12X) as well as physical and/or mental impairment (7X). The achieved participation rate is in line with reported response rates in patient satisfaction studies.*^7^

**How did those who agreed differ from those who did not agree?**

*As stated in the `results section`, mean age, gender and clinical WFNS scores of SAH patients did not differ significantly between study participants (n=93) and non-responders (n=52).*

**Describe and justify the methods and tests used for data analysis.**

*We solely used pain and health-related quality of life surveys which have been thoroughly evaluated regarding their psychometric properties in previous studies and utilized in a broad spectrum of pain conditions*

1. **Present the results of the research. The results section should be clear, factual, and concise.**

*The study results are presented clearly and concise in the `results section`.*

1. **Interpret and discuss the findings.**

*The main findings are put into the scientific context. The `discussion section` comprises a critical reflection upon study results including methodological limitations.*

1. **Present conclusions and recommendations.**

*Conclusions and recommendations are presented concisely in the `conclusion section` of the main document.*

**Sources**

1. Kelley K. Good practice in the conduct and reporting of survey research. *Int J Qual Health Care*. 2003;15(3):261-266. doi:10.1093/intqhc/mzg031

2. Nagel B, Gerbershagen HU, Lindena G, Pfingsten M. [Development and evaluation of the multidimensional German pain questionnaire]. *Schmerz Berl Ger*. 2002;16(4):263-270. doi:10.1007/s00482-002-0162-1

3. Bauer B, Evers S, Gralow I, Husstedt IW. [Psychosocial handicap due to chronic headaches. Evaluation of the inventory of Disabilities caused by Headache]. *Nervenarzt*. 1999;70(6):522-529.

4. Melzack R. The short-form McGill Pain Questionnaire. *Pain*. 1987;30(2):191-197.

5. Ware J, Kosinski M, Keller SD. A 12-Item Short-Form Health Survey: construction of scales and preliminary tests of reliability and validity. *Med Care*. 1996;34(3):220-233.

6. Nilges P, Essau C. [Depression, anxiety and stress scales: DASS--A screening procedure not only for pain patients]. *Schmerz Berl Ger*. 2015;29(6):649-657. doi:10.1007/s00482-015-0019-z

7. Sitzia J, Wood N. Response rate in patient satisfaction research: an analysis of 210 published studies. *Int J Qual Health Care J Int Soc Qual Health Care*. 1998;10(4):311-317. doi:10.1093/intqhc/10.4.311
